# Orthohantaviruses belonging to three phylogroups all inhibit apoptosis in infected target cells

**DOI:** 10.1038/s41598-018-37446-1

**Published:** 2019-01-29

**Authors:** Carles Solà-Riera, Shawon Gupta, Hans-Gustaf Ljunggren, Jonas Klingström

**Affiliations:** 1 Department of Medicine Huddinge, Center for Infectious Medicine, Karolinska Institutet, Karolinska University Hospital, Stockholm, Sweden; 2 Department of Infectious Diseases, Virology, University Hospital Heidelberg, Heidelberg, Germany

## Abstract

Orthohantaviruses, previously known as hantaviruses, are zoonotic viruses that can cause hantavirus pulmonary syndrome (HPS) and hemorrhagic fever with renal syndrome (HFRS) in humans. The HPS-causing Andes virus (ANDV) and the HFRS-causing Hantaan virus (HTNV) have anti-apoptotic effects. To investigate if this represents a general feature of orthohantaviruses, we analysed the capacity of six different orthohantaviruses – belonging to three distinct phylogroups and representing both pathogenic and non-pathogenic viruses – to inhibit apoptosis in infected cells. Primary human endothelial cells were infected with ANDV, HTNV, the HFRS-causing Puumala virus (PUUV) and Seoul virus, as well as the putative non-pathogenic Prospect Hill virus and Tula virus. Infected cells were then exposed to the apoptosis-inducing chemical staurosporine or to activated human NK cells exhibiting a high cytotoxic potential. Strikingly, all orthohantaviruses inhibited apoptosis in both settings. Moreover, we show that the nucleocapsid (N) protein from all examined orthohantaviruses are potential targets for caspase-3 and granzyme B. Recombinant N protein from ANDV, PUUV and the HFRS-causing Dobrava virus strongly inhibited granzyme B activity and also, to certain extent, caspase-3 activity. Taken together, this study demonstrates that six different orthohantaviruses inhibit apoptosis, suggesting this to be a general feature of orthohantaviruses likely serving as a mechanism of viral immune evasion.

## Introduction

Orthohantaviruses, of the order *Bunyavirales* and previously known as hantaviruses, are small single-stranded negative-sense RNA viruses with a tri-segmented genome (S, M and L segments) encoding four to five proteins. The S segment encodes a nucleocapsid protein (N), the M segment two glycoproteins (Gn and Gc), and the L segment an RNA dependent RNA polymerase^1–5^. Additionally, the S segment of some orthohantaviruses also encodes a non-structural protein called NSs^5^. The natural hosts for orthohantaviruses are various small animals, mainly rodents, but also moles, shrews and bats, and as recently shown fishes and reptiles too^1–6^. Each distinct orthohantavirus primarily infects one specific animal species^7,8^. Orthohantaviruses establish life-long infection in their respective natural hosts^2,5^. However these viruses cause strong immune responses in the natural host^9^ and it is currently not well known how orthohantaviruses avoid being eradicated.

Orthohantaviruses have a worldwide distribution^4,5^. At present more than fifty different orthohantaviruses, whereof twenty are pathogenic to humans, have been identified^2^. Rodent-borne orthohantaviruses can cause hantavirus pulmonary syndrome (HPS; also known as hantavirus cardiopulmonary syndrome (HCPS)) and hemorrhagic fever with renal syndrome (HFRS)^1–3^. Three different rodent subfamilies – *Arvicolinae*, *Sigmodontinae* and *Murinae* – harbor the majority of the known orthohantaviruses, including all known HPS- and HFRS-causing viruses as well as several non-pathogenic ones. Phylogenetic analyses have shown that *Arvicolinae*-borne and *Sigmodontinae-*borne orthohantaviruses are closely related to each other. In contrast, orthohantaviruses carried by *Murinae* rodents cluster with certain mole- (*Talpidae*) and shrew-borne (*Soricidae*) orthohantaviruses which to date have not been associated with human disease^10^. *Sigmodontinae*-borne orthohantaviruses (*e.g*. Andes virus (ANDV) and Sin Nombre virus (SNV)) are found in the Americas. In contrast, the vast majority of *Murinae*-borne orthohantaviruses (such as Hantaan virus (HTNV), Seoul virus (SEOV) and Dobrava virus (DOBV)) as well as *Arvicolinae-*borne orthohantaviruses (*e.g*. Puumala virus (PUUV) and Tula virus (TULV)) have been identified in Euroasia^2,10^.

The highly pathogenic ANDV and SNV cause HPS in South and North America respectively with mortality rates of 35–40%^3,11,12^. HTNV causes HFRS in Asia^1–5,13^, whereas SEOV causes HFRS worldwide^1–5,14,15^. PUUV stands as the main contributor to HFRS in Europe^4,16,17^, while DOBV is the causative agent of the most severe HFRS cases in central Europe^18,19^. Although mortality rates are generally lower for the Euroasian orthohantaviruses, some of them, such as DOBV, are associated with mortality rates of more than 10%^19^. Many other orthohantaviruses, such as the *Arvicolinae*-borne Prospect Hill virus (PHV) and TULV, are believed not to cause disease in immune-competent individuals^20,21^. Capillary leakage, strong immune activation and inflammation are common hallmarks of both HPS and HFRS^1–4^. Endothelial cells represent the main target for orthohantaviruses^22^; interestingly though, orthohantaviruses *per se* do not cause any direct cytopathic effects^23,24^ and despite the robust immune activation observed in patients, infected endothelial cells remain undamaged^25–27^.

Apoptosis is a well-regulated mechanism to eliminate cells, including virus-infected cells or tumorigenic cells. Apoptosis plays an important role in restricting the dissemination of pathogens, such as viruses, throughout the body. Caspases (cysteine-dependent aspartate-directed proteases) act as main orchestrators of apoptosis. These proteases are present as inactive zymogens requiring cleavage and subsequent oligomerization to become active. During apoptosis, caspase-3 is activated and cleaves several cellular key protein components, such as the poly ADP-ribose polymerase (PARP)^28,29^. Caspase-3 is necessary for chromatin condensation and DNA fragmentation, two typical hallmarks of apoptosis^30^. Because of the crucial role played in determining cell fate, the action of caspases is regulated at multiple levels, both prior to and after activation^31–33^. Given the importance of apoptosis-inducing pathways in cellular anti-viral defense, it is not surprising that some viruses have been shown to interfere with one or more components of these pathways^33–40^.

Cytotoxic lymphocytes, such as natural killer (NK) cells and cytotoxic T cells (CTL), represent important components of the immune response towards virus infections. Both cell types kill virus-infected cells in a similar manner, mainly via cytotoxic granule-mediated activation of target cell apoptosis. The cytotoxic granules contain granzymes, which upon release into target cells cleave certain cellular substrates thereby activating cell death pathways^41^. Mainly, this occurs via direct granzyme B activation of caspase-3^42,43^. Granzyme B has also been reported to induce programmed cell death in a caspase-independent manner^41,44^. Orthohantavirus-infected patients show robust cytotoxic lymphocyte responses encompassing a long-lived NK cell response including specific expansion of NKG2C^+^ NK cells^45^ and strong virus-specific cytotoxic CD8^+^ T cell responses at onset of disease^46–49^, suggesting that cytotoxic lymphocytes play important roles in human orthohantavirus infections^50^.

We recently showed that ANDV and HTNV confer resistance to cytotoxic lymphocyte-mediated killing of infected endothelial cells^51^. In the present study, we aimed at defining if the anti-apoptotic features of ANDV and HTNV represent a common attribute shared by other pathogenic orthohantaviruses.

## Results

### Orthohantaviruses protect infected cells from staurosporine-induced apoptosis

**Figure 1:**
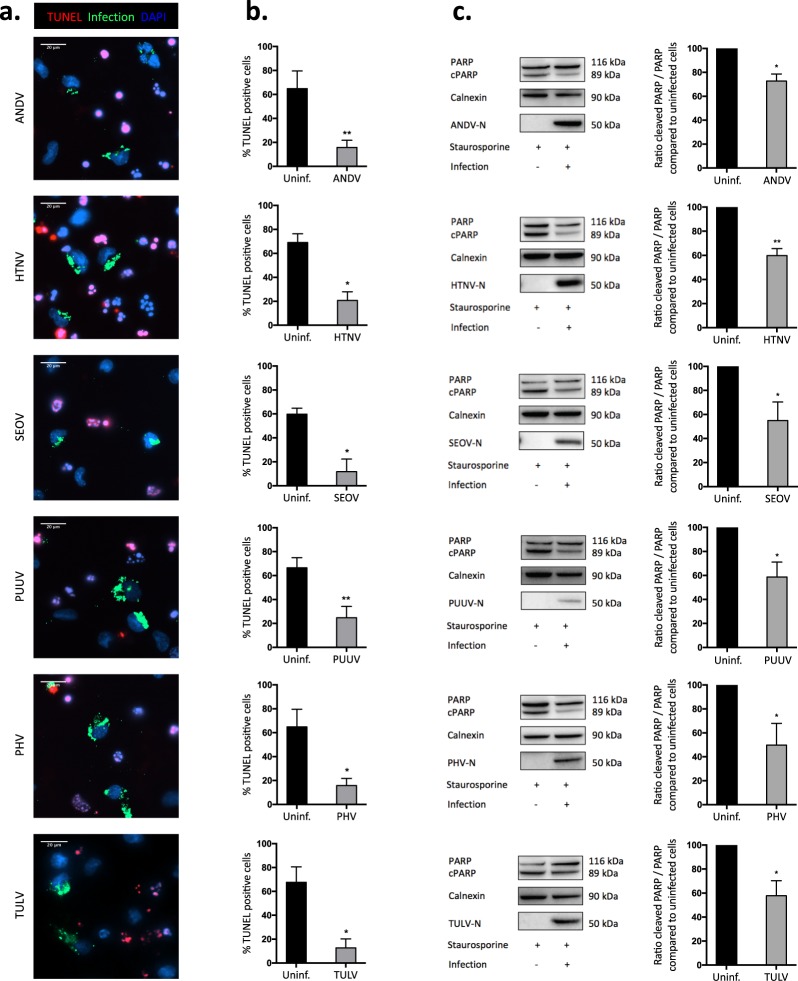
Orthohantaviruses hinder staurosporine-induced apoptosis in human primary endothelial cells. (**a**) Representative immunofluorescence images from three independent experiments of ANDV-, HTNV-, SEOV-, PUUV-, PHV- and TULV-infected HUVEC after exposure to staurosporine (2 μM) for approximately 4 hours. Cells were infected at a low MOI (0.01) to achieve 20 to 30% infection at 4 days post-infection. Following staurosporine-treatment, cells were stained with TUNEL (red) to assess apoptosis, convalescent PUUV patient serum (green) to detect virus infection and DAPI (blue) for nuclear counterstaining. Scale bar, 20 μm. (**b**) Graphs showing TUNEL-positive uninfected and infected cells. Data shown represent the mean ± SD of three independent experiments. (**c**) Western blot analyses of PARP (full-length and cleaved) in total cell lysate from infected and uninfected cells exposed to staurosporine (2 μM) for approximately 4 hours or until observing the effects of staurosporine in treated control cells. MOI 1 of virus was used, resulting in ≥95% of the cells being infected 4 days after infection (Supplementary Fig. 1). Specific monoclonal antibodies (mabs *1C12* for ANDV, HTNV, SEOV, PUUV and TULV, and *7B3F7* for PHV) were used for detection of N protein. Calnexin was used as loading control. One representative experiment out of three independent experiments is shown. Band intensity was analysed by densitometry of caspase-3 cleaved PARP and full-length PARP. The ratios in intensity between cleaved and full-length PARP were calculated and compared between infected and uninfected conditions. The ratio from uninfected cells represents maximal caspase-3 cleaved PARP/full-length PARP ratio (100%). The graphs show mean ± SD of the ratio between cleaved and full-length PARP from three experiments. Paired t-test: *p < 0.05; **p < 0.01.

To test if different pathogenic and non-pathogenic orthohantaviruses from different phylogroups could inhibit apoptosis, we infected cells at MOI of 0.01 in order to achieve 20 to 30% infection rate at four days post-infection. Cells were then treated with the apoptosis-inducing chemical staurosporine. Apoptosis was then assessed by TUNEL, and percentage of apoptotic infected and non-infected cells on the same slide determined (Fig. 1a,b). Inhibition of apoptosis was observed for ANDV (76.2 ± 3.3% (mean ± SEM) less apoptosis compared to uninfected cells), HTNV (69.5 ± 6.7% less apoptosis), SEOV (79.4 ± 11.3% less apoptosis), PUUV (63.2 ± 6.0% less apoptosis), PHV (76.2 ± 3.3% less apoptosis), and TULV (80.6 ± 6.6% less apoptosis) (Fig. 1b). Accordingly, in cell cultures with high infectivity rates (i.e. ≥95% of the cells being infected four days after infection (Supplementary Fig. 1)), the same results were obtained after staurosporine treatment. Orthohantavirus-infected cultures showed significantly lower apoptotic cells as observed by TUNEL, when compared to the uninfected cultures (Supplementary Fig. 2).

**Figure 2:**
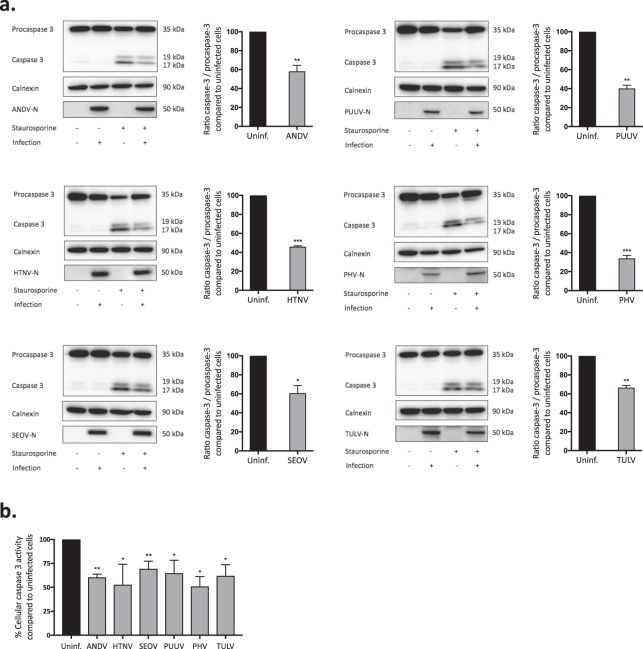
Orthohantavirus infection hampers the processing of caspase-3 into its active form and inhibits cellular caspase-3 activity. (**a**) Western blot analyses of caspase-3 in total cell lysate from staurosporine-exposed infected and uninfected HUVEC. MOI 1 of virus was used, and cells were treated with 2 μM staurosporine for approximately 4 hours. N protein was detected using the monoclonal antibodies *1C12* for ANDV, HTNV, SEOV, PUUV and TULV, and *7B3F7* for PHV. One representative experiment out of three is shown. Band intensity was analysed by densitometry of cleaved caspase-3 and total caspase-3. A ratio was then calculated and compared between infected and uninfected conditions. Staurosporine-exposed uninfected cells represent maximal cleaved caspase-3/full-length caspase-3 ratio. The graphs show mean ± SD of three independent experiments. (**b**) Caspase-3 activity in infected A549 cells after exposure to 2 μM staurosporine for 4 hours. ≥95% of the A549 cells were infected as assessed by immunofluorescence (Supplementary Fig. 1). Cellular caspase-3 activity in infected cells was compared to that in uninfected cells at the same time point. Total protein levels in the samples were measured using a Bradford assay. Staurosporine-treated uninfected cells represent maximal caspase-3 activity/mg of total cellular protein. Results show mean ± SD of three independent experiments. Paired t-test: *p < 0.05; **p < 0.01; ***p < 0.001.

To verify the results observed using the TUNEL assay, we analyzed for levels of caspase-cleaved poly ADP-ribose polymerase (PARP) and caspase-3 activity in staurosporine-treated cells. To that purpose cells were once more infected at MOI 1, resulting in ≥95% of the cells being infected four days after infection (Supplementary Fig. 1). Then infected and non-infected cell cultures as control were exposed to staurosporine. Compared to uninfected cells, the cleavage of PARP was clearly hampered in cells infected with the different orthohantaviruses (Fig. 1c). The observed inhibition of PARP-cleavage in infected cells indicated that orthohantaviruses in general, as previously reported for ANDV^51^, interfere with activation and/or function of caspase-3. Indeed, less cleaved, activated, caspase-3 was observed in infected compared to uninfected cells (Fig. 2a), showing that all tested orthohantaviruses inhibit staurosporine-mediated activation of caspase-3. In line with this finding, analysis of total cellular caspase-3 activity revealed significantly reduced caspase-3 activity in staurosporine-exposed infected, compared to uninfected, cells (Fig. 2b).

### Orthohantaviruses inhibit cytotoxic lymphocyte-mediated killing of infected cells

**Figure 3:**
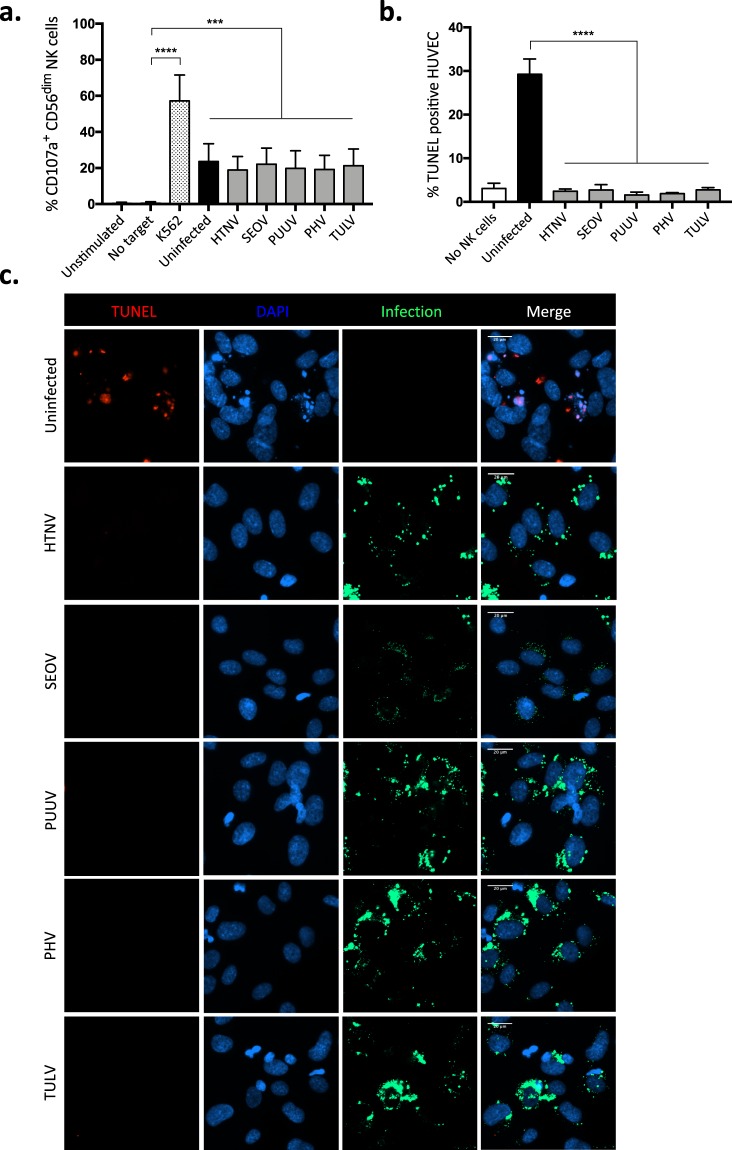
Orthohantaviruses protect infected endothelial cells from NK cell-mediated killing. (**a**) Expression of CD107a on IL-15 activated CD56^dim^ NK cells not exposed to target cells or exposed to orthohantavirus-infected or uninfected, HLA-blocked, endothelial cells for 2 hours. K562 cells were used as a positive control of NK cell degranulation. Frequencies shown represent mean ± SD of three independent experiments of three blood donors in each (n = 9). Paired t-test: *p < 0.05; **p < 0.01; ***p < 0.001; ****p < 0.0001. (**b**) TUNEL positive cells after exposure to NK cells. Data represent mean ± SD from three independent experiments with a total of nine donors. One-way ANOVA: *p < 0.05; **p < 0.01; ***p < 0.001; ****p < 0.0001. (**c**) Representative TUNEL stained immunofluorescence images of infected and uninfected primary endothelial cells after co-incubation with NK cells. Endothelial cells were exposed to NK cells at a 1:1 effector to target ratio (E:T ratio). Target cells were stained using TUNEL (red), convalescent PUUV patient serum (green) and DAPI for nuclear counterstaining (blue). Immunofluorescence images are representative data from nine different NK cell donors in total. Scale bar, 20 μm.

We next tested if the six orthohantaviruses differed in their capacity to inhibit cytotoxic lymphocyte-mediated apoptosis. To this end, we used highly cytotoxic IL-15 activated NK cells. First, we assessed if any of the orthohantaviruses affected NK cell degranulation towards HLA class I-blocked target cells. Increased cell surface expression levels of CD107a were detected on NK cells after co-incubation with the different orthohantavirus-infected as well as uninfected cells, showing that none of the tested orthohantaviruses affected the degranulation capacity of NK cells towards HLA class I-blocked cells (Fig. 3a; Supplementary Fig. 3). We next assessed the level of apoptosis in HLA class I-blocked endothelial cells after co-incubation with activated NK cells. Strikingly, while NK cells efficiently killed uninfected cells, all orthohantaviruses efficiently protected infected cells from NK cell killing, suggesting that inhibition of lymphocyte-mediated cytotoxicity is a general feature of orthohantaviruses (Fig. 3b,c).

### The N protein of different orthohantaviruses contains cleavage sites for caspase-3 and granzyme B

**Figure 4:**
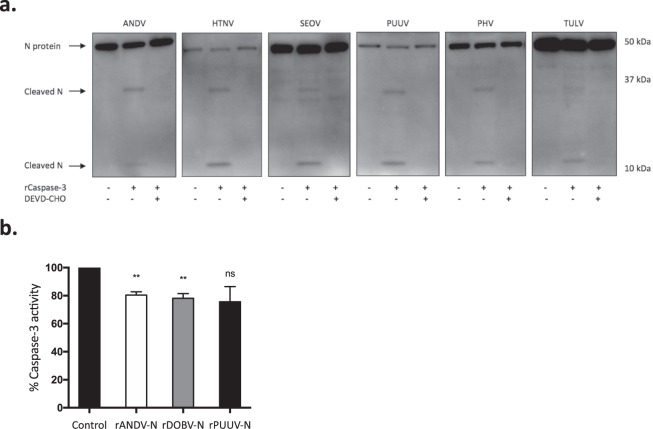
Orthohantaviruses N protein contains cleavage sites for caspase-3 and weakly inhibits caspase-3 activity. (**a**) Western blot analyses showing two cleaved fragments of the different orthohantavirus N protein after incubation with or without recombinant caspase-3 in the presence or absence of the caspase-3-inhibitor Ac-DEVD-CHO. The monoclonal antibody 7A2/D5 was used in order to detect the cleaved fragments of N protein. One representative experiment out of three is shown. (**b**) Caspase-3 activity after incubation with recombinant ANDV, PUUV or DOBV N protein (rANDV-N, rPUUV-N or rDOBV-N), or with recombinant control protein (DHFR). Data shown represent mean ± SD of three independent experiments. Paired t-test: ns non-significant; *p < 0.05; **p < 0.01.

**Figure 5:**
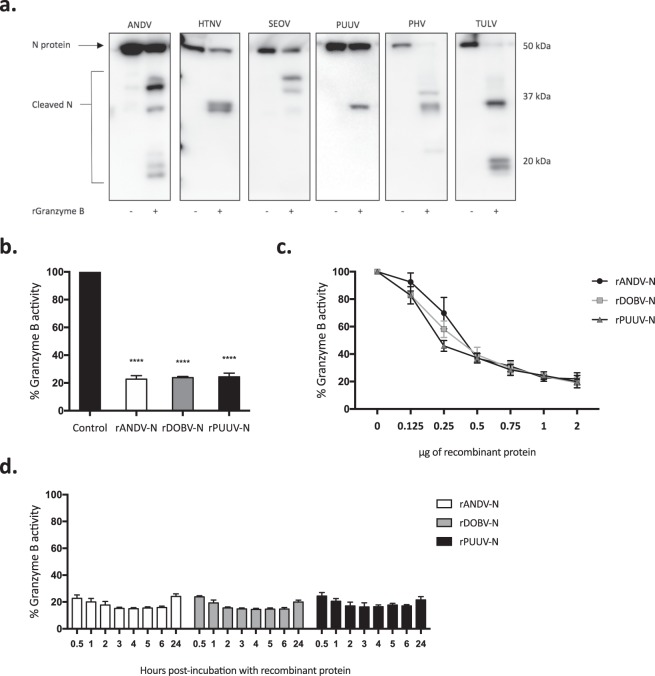
N protein of different orthohantaviruses contain cleavage sites for granzyme B and robustly inhibit the enzymatic activity of granzyme B. (**a**) Western blot analyses showing cleaved fragments of the different orthohantavirus N protein after incubation with or without recombinant granzyme B. The monoclonal antibodies 7A2/D5, 7B3/F7 and 8F3/F8 were used in order to detect the cleaved fragments of N protein. Data shown represent one experiment out of three. (**b**) Granzyme B activity after 30 minutes of incubation with rANDV-N, rPUUV-N or rDOBV-N. The graphs show mean ± SD of three independent experiments. (**c**) Granzyme B activity after 30 minutes of incubation with increasing amounts of rANDV-N, rPUUV-N or rDOBV-N. DHFR was added to the different samples in order to adjust the total amount of protein to 2 μg. The results shown represent mean ± SD of three independent experiments. (**d**) Granzyme B activity was measured at different time-points after incubation with recombinant N protein at 37 °C – 0.5, 1, 2, 3, 4, 5, 6 and 24 hours. Data shown represent mean ± SD of three independent experiments. Paired t-test: ns non-significant; *p < 0.05; **p < 0.01; ***p < 0.001; ****p < 0.0001.

The N protein of orthohantaviruses is highly expressed in the cytoplasm of infected cells^22^. We have previously shown that ANDV N protein interacts with, and inhibits, caspase-3 and granzyme B^51^. Incubation of viral proteins with recombinant active human caspase-3 revealed that there are potential caspase-3 cleavage sites in the N protein of all tested orthohantaviruses (Fig. 4a). A common cleavage pattern was observed, showing two different caspase-3 cleavage products of the N protein, with molecular weights of approximately 35 kDa and 10 kDa. The cleaved products were not observed in the presence of the caspase-3 inhibitor Ac-DEVD-CHO (Fig. 4a). Incubation of viral proteins with recombinant active human granzyme B showed the presence of several potential granzyme B cleavage sites in the evaluated N proteins (Fig. 5a). However, the cleavage led to many distinct fragments, and in contrast to the common cleavage pattern observed for caspase-3-mediated cleavage of the N protein (Fig. 4a), granzyme B-mediated cleavage resulted in unique cleavage patterns for the specific N proteins (Fig. 5a).

### The N protein of different orthohantaviruses can inhibit caspase-3 and granzyme B activity

We next analyzed the potential caspase-3 inhibitory capacity of the N proteins from three different orthohantaviruses representing the three distinct subfamilies – ANDV (*Sigmodontinae*-borne orthohantavirus), DOBV (*Murinae*-borne orthohantavirus) and PUUV (*Arvicolinae*-borne orthohantavirus). Co-incubation of the recombinant N proteins (rANDV-N, rPUUV-N or rDOBV-N) with recombinant active human caspase-3 resulted in a slight inhibition of caspase-3 activity (Fig. 4b), suggesting that orthohantaviruses belonging to all three distinct subfamilies may inhibit caspase-3 activity. Strikingly, despite the clear differences observed in the granzyme B proteolytic processing of different orthohantaviruses N protein (Fig. 5a), rANDV-N, rDOBV-N, and rPUUV-N all of them strongly inhibited granzyme B activity (Fig. 5b–d), suggesting that orthohantavirus N protein is a potent inhibitor of granzyme B.

## Discussion

Many viruses can hijack the cellular machinery to inhibit apoptosis or other host antiviral responses, thereby enabling successful viral replication, often without causing direct cytopathic effects^52^. We here report that orthohantaviruses belonging to three different phylogroups, representing both pathogenic and non-pathogenic orthohantaviruses, all strongly inhibit both staurosporine-induced and cytotoxic lymphocyte-mediated apoptosis in infected endothelial cells.

Cytotoxic lymphocytes, such as NK cells and CTLs, are specialized lymphocyte subsets capable of eliminating target cells by inducing apoptosis in them. The finding that in addition to ANDV and HTNV^51^, the HFRS-causing SEOV and PUUV, and the non-pathogenic PHV and TULV, also protect infected cells from cytotoxic lymphocyte-mediated killing, suggests that resistance to cytotoxic lymphocyte-mediated apoptosis is a common feature of orthohantaviruses, potentially contributing to their capacity to cause life-long infection in natural hosts. Moreover, these data provide a possible explanation for the observation that in HCPS and HFRS patients, despite the overwhelming cytotoxic lymphocyte responses, infected endothelial cells remain undamaged^22,26,27,45,47,48]^.

All tested orthohantaviruses inhibited staurosporine-induced apoptosis. Orthohantavirus replication is slower than most other RNA viruses^53,54^, and no cytopathic effects are observed in infected cells^23–25,55^. As such, viral interference with apoptosis can be critical for the successful production of progeny viruses, especially at late stages after infection. We observed lower levels of cellular caspase-3 activity in staurosporine-treated infected compared to uninfected cells, suggesting that orthohantaviruses interfere with staurosporine-induced apoptosis upstream of caspase-3 activation. During orthohantavirus-infection the N protein is highly expressed in endothelial cells^22,56^. Caspase-3 and granzyme B both cleave after aspartic acid in targeted proteins, but potential cleavage sites often differ for the two enzymes^57^. The N protein of the different orthohantaviruses were all cleaved by caspase-3 and granzyme B, suggesting that these proteases can interact with the N protein. Further, ANDV, PUUV and DOBV recombinant N protein all slightly inhibited caspase-3 activity, and strongly inhibited granzyme B activity, suggesting that the N protein has anti-apoptotic effects. Similar results on caspase-3 and granzyme B have been observed for proteins encoded by certain other viruses with anti-apoptotic effects: poxviruses and baculoviruses encode potent inhibitors such as CrmA, L4-100K, and p35^34–36,58–63^, while Epstein-Barr virus and adenoviruses produce proteins that mimic anti-apoptotic cellular proteins^37,38^. However, these are DNA viruses encoding for up to 200 proteins, to our knowledge orthohantaviruses are the first RNA viruses shown to be able to inhibit these proteases.

Besides orthohantaviruses, other emerging, hemorrhagic fever-causing, RNA viruses such as Lassa virus, Ebola virus and Junin virus are also able to establish persistent infections within their host^52,64–66^. Importantly, the molecular mechanisms allowing for these RNA viruses to establish persistent infection, and why the immune system fails to clear these infections, are largely unknown. If inhibition of apoptosis is a common feature of different emerging zoonotic RNA viruses remains to be investigated. In conclusion, we show that orthohantaviruses display a robust anti-apoptotic capacity. This finding suggests a possible role for orthohantavirus-mediated apoptosis inhibition in persistent infection of natural hosts and in pathogenesis.

## Materials and Methods

### Cells and viruses

Primary human umbilical vein endothelial cells (HUVEC) were purchased form Lonza. The cells were grown in EGM media supplemented with growth factors according to manufacturer’s instructions (Lonza). During infection and co-culture experiments, HUVEC were grown without hydrocortisone. The human lung epithelial cell line A549 (American Type Culture Collection (ATCC) CLL-185) was grown in MEM supplemented with 5% FBS, 100 U/mL of penicillin, and 100 μg/mL of streptomycin (all from Thermo Fisher Scientific). K562 cells (ATCC CCL-243) were grown in complete RPMI 1640 medium (Invitrogen; supplemented with 10% FBS, 100 μg/mL L-glutamine, 100 U/mL penicillin and 100 μg/mL streptomycin). Buffy coats from healthy donors were obtained from the Blood Transfusion Clinic at the Karolinska University Hospital Huddinge in Stockholm, Sweden (ethical permit from Stockholm ethical review board: 2016/1415-32). Peripheral blood mononuclear cells (PBMC) were isolated by density centrifugation (Ficoll-Hypaque from GE Healthcare) and NK cells were subsequently purified using a negative NK cell isolation kit (Miltenyi Biotec) resulting in >95% pure NK cells (data not shown). NK cells were cultured overnight in complete RPMI 1640 medium, alone or supplemented with IL-15 (20 ng/mL) from R&D Systems. ANDV strain Chile-9717869, HTNV strain 76–118^13^, SEOV strain R22, PUUV strain Kazan-E6^67^, PHV strain PH-1, TULV strain Moravia/Ma5302V/94^68^ were propagated and titrated as earlier described^69^. All experiments involving live orthohantaviruses were conducted in a biosafety level-3 laboratory.

### Chemicals, antibodies and other reagents

Detection of orthohantavirus-infected cells was performed using convalescent human serum followed by secondary staining with FITC-conjugated, goat anti-human IgG (Sigma-Aldrich) or AF594-conjugated, goat anti-human IgG (Thermo Fisher Scientific). Nuclei counterstaining was achieved using 4′,6′-diamidino-2-phenylindole (DAPI; Sigma-Aldrich). For immunoblotting, the monoclonal antibodies (mAbs) 1C12, 7A2/D5, 7B3/F7 and 8F3F8, specific for orthohantavirus nucleocapsid protein, were used as previously described^70,71^. MAbs against PARP (#9532) and calnexin (#2679) were purchased from Cell Signaling Technology, while anti-caspase-3 antibody (#ab13585) was from Abcam. Staurosporine and 2X Reaction Buffer were from BioVision. Recombinant human caspase-3 and granzyme B were from BioVision and from BD Biosciences, respectively, and the caspase-3 inhibitor Ac-DEVD-CHO was from Sigma-Aldrich. Recombinant ANDV-N protein was kindly provided by Dr. N. Tischler, and prepared as earlier described^70^. The recombinant DOBV-N strain Slovenia and PUUV-N strain Vranica proteins were purchased from Abcam (#ab74556; #ab74555).

### Orthohantavirus infection of endothelial cells

Infection was performed after diluting the virus stocks to the appropriate concentration in EGM medium (Lonza) for HUVEC, or MEM medium for A549 cells. The virus dilution was then added to cells when these had reached 70% confluence for HUVEC or 100% for A549 cells, and incubated for 1 hour at 37 °C. After 1 hour, the virus inoculum was removed and fresh pre-warmed medium was added to the cells. For TUNEL analysis of cells exposed to staurosporine, a low multiplicity of infection (MOI) of 0.01 was used, resulting in around 20 to 30% of the cells being infected 96 hours post-infection (p.i.). In other experiments, infection was performed at MOI 1, reaching infection rates ≥95% at 96 hours p.i.

### Staurosporine killing assay

Staurosporine was diluted in EGM medium to a final concentration of 2 μM. Cells were incubated with the diluted chemical for approximately 4 hours at 37 °C, before being harvested for further analysis.

### Immunofluorescence assay

Cells grown on coverslips were fixed with methanol for 10 minutes. Specimens were then blocked with blocking solution (PBS containing 5% normal goat serum and 0.3% Triton X-100) for 1 hour. The samples were incubated with primary antibody for 1 hour at RT. After washing with PBS, the corresponding secondary antibody was added for 1 hour at RT. DAPI was used for nuclei counterstaining. The coverslips were washed with PBS and then mounted on glass slides using ProLong Gold Antifade Mountant (Thermo Fisher Scientific).

### TUNEL assay

Cells were grown on coverslips and fixed with 4% PFA at RT, for 20 minutes and then washed twice with PBS. Cells were then permeabilized with 0.5% Triton X-100 in PBS for 8 minutes at 4 °C. TUNEL (Terminal deoxynucleotidyl transferase-mediated dUTP nick-end labeling) was executed as previously described^72^, using the *in situ* cell death detection kit, TMR red (Roche). After the TUNEL reaction, cells were stained for infection and the nuclei counterstained following the immunofluorescence staining procedure as described above.

### Immunoblotting

For Western blot experiments, cells were harvested and centrifuged at 1500 rpm for 5 minutes at 4 °C. Afterwards, the cells were washed once with ice-cold PBS. Cell pellets were resuspended in lysis buffer (150 mM NaCL, 2 mM EDTA, 1% NP-40, and 50 mM Tris (pH 7.6)) complemented with protease and phosphatase inhibitors (cOmplete mini cocktail tablets and PhosSTOP inhibitor tablets (Roche)), according to manufacturer’s guidelines. Samples were stored at −80 °C until further analysis, or directly mixed 3:1 with NuPAGE LDS sample buffer (Thermo Fisher Scientific) supplemented with 2.5% 2-mercaptoethanol and incubated at 96 °C for 10 min. The samples were run on NuPAGE Novex Bis-Tris protein gels (Thermo Fisher Scientific) and transferred to PVDF membranes using the iBlot2 Gel Transfer Device (Thermo Fisher Scientific). Blocking of the membranes was done at RT for 1 hour in PBS containing 5% non-fat powdered milk and 0.2% Tween 20 (Sigma-Aldrich). Subsequently, the membranes were incubated with primary mAbs overnight, at 4 °C, followed by the addition of horseradish peroxidase-conjugated anti-mouse or anti-rabbit IgG (BioRad). Readout was obtained using ECL Plus Western blotting detection kit (GE Healthcare Life Sciences), following manufacturer’s instructions. Images were acquired with an MF-ChemiBIS 3.2 (DNR BioImaging Systems Ltd.). Stripping of membranes was performed in Re-blot Plus mild antibody stripping solution (Merck Millipore), following the manufacturer’s guidelines.

### Virus nucleocapsid protein cleavage assay

Viruses, heat-inactivated at 96 °C for 10 minutes, were incubated with active human recombinant caspase-3 (with or without caspase-3 inhibitor) or active human recombinant granzyme B, in reaction buffer (BioVision) at 37 °C for 1 hour or as stated in the text. Samples were then processed for immunoblotting as described above to analyze for cleavage of N protein.

### Enzymatic activity assays

The enzymatic activity of caspase-3 and granzyme B were assessed using specific activity assays according to manufacturers’ instructions (Sigma-Aldrich for caspase-3 and Calbiochem for granzyme B). For analysis of cellular caspase-3 activity after staurosporine-treatment, cells were harvested and centrifuged at 1500 rpm for 5 minutes at 4 °C. Afterwards, the cells were washed once with ice-cold PBS. Cell pellets were resuspended in lysis buffer (150 mM NaCL, 2 mM EDTA, 1% NP-40, and 50 mM Tris (pH 7.6)) without protease inhibitors and snap frozen in liquid nitrogen. Total protein concentration in samples was analyzed by Bradford assay. In order to study the inhibitory effect of N protein, active human recombinant caspase-3 or active human recombinant granzyme B (0.1 μg) was mixed with reaction buffer and 1 μg of recombinant N protein or 1 μg of rDHFR as control, and incubated at 37 °C for 30 minutes or as stated in the text. The assays were performed at a total volume of 100 μL. For analysis of the effect of increasing concentrations of recombinant N protein on granzyme B activity, DHFR was added to the different samples in order to adjust the total amount of protein to 2 μg.

### Co-incubation of NK cells with endothelial cells

NK cells were stimulated overnight with IL-15 (20 ng/mL; R&D Systems). To allow for maximal NK cell killing capacity, HLA class I molecules were blocked on target endothelial cells using Dx17 mAbs (BD Biosciences) for 30 minutes at 37 °C. The activated NK cells were then, at an effector:target (E:T) ratio of 1:1, co-incubated with the endothelial cells at 37 °C for 2 hours or as stated in the text.

### Flow cytometry

Analyses of NK cell activation and degranulation were performed after co-incubation with target cells. Briefly, NK cells were stained in FACS buffer (PBS containing 2% FBS and 2 mM EDTA) for 20 minutes at RT in the dark, then washed twice with FACS buffer and fixed in 2% PFA for 10 minutes at RT in the dark. Samples were acquired on an LSRFortessa (BD Biosciences) and data was analyzed using the software FlowJo version 9.8.1 (Tree Star Inc.). The following mAbs were used for flow cytometry: CD107a (clone H4A3, VioBright-FITC) from Miltenyi Biotec, CD3 (clone UCHT1, AF700), CD14 (clone M5E2, V500), CD19 (clone HIB19, V500), CD16 (clone 3G8, BV711), CD69 (clone FN50, PE-CF594), CD56 (clone B159, PE-Cy7), all from BD Biosciences. A cell death marker (LIVE/DEAD Fixable Aqua Dead Cell Stain Kit from Thermo Fisher Scientific) was used to label dead cells.

### Cell count and infection quantification

Quantification of total cell numbers, TUNEL positive cells and infected cells was done manually and by employing the open source software ImageJ (Imaging Processing and Analysis in Java; http://rsb.info.nih.gov/ij).

### Densitometry

The membranes were scanned and then the intensity of bands was quantified using ImageJ.

### Statistical methods

The paired T-test and one-way ANOVA test were used for statistical analysis. The analysis wes performed using GraphPad Prism software version 7.0 (GraphPad Software Inc.). Statistical significance is symbolized with asterisks (ns, non-significant; *p < 0.05, **p < 0.01, ***p < 0.001, ****p < 0.0001).

## Supplementary information

Supplementary Dataset 1
