# Supplementary material for: Orthohantaviruses belonging to three phylogroups all inhibit apoptosis in infected target cells
Source: Sci Rep. 2019 Jan 29;9:834. doi: 10.1038/s41598-018-37446-1 (PMC6351540; doi:10.1038/s41598-018-37446-1)
Supplement: Supplementary file 1 — Supplementary Dataset 1 [file 41598_2018_37446_MOESM1_ESM.pdf]

Orthohantaviruses belonging to three phylogroups all inhibit apoptosis in infected target cells

Carles Solà-Riera[1], Shawon Gupta[1,2], Hans-Gustaf Ljunggren[1], and Jonas Klingström[1]

[1]Department of Medicine Huddinge, Center for Infectious Medicine, Karolinska Institutet, Karolinska University Hospital, Stockholm, Sweden.

[2]Department of Infectious Diseases, Virology, University Hospital Heidelberg, Heidelberg, Germany.

Supplementary Figure 1.

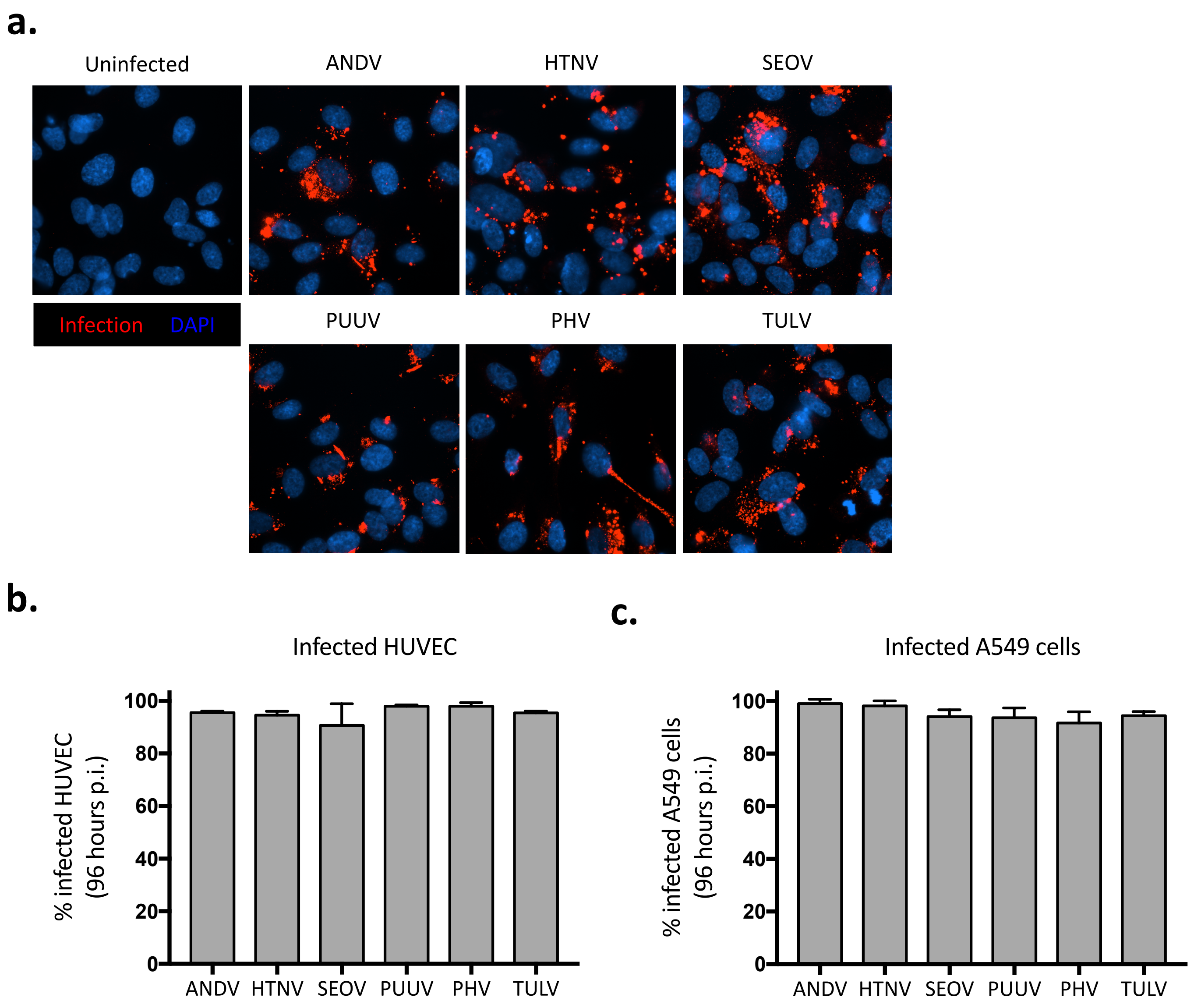

Supplementary Figure 1. Infection rates on endothelial cells. (a) Representative immunofluorescence images of ANDV-, HTNV-, SEOV-, PUUV-, PHV- and TULV-infected HUVEC 4 days after infection. HUVEC were infected at MOI 1. Cells were fixed 4 days later and stained with convalescent PUUV patient serum to detect viral proteins (red) and DAPI for nuclear counterstaining (blue). Images are representative from three independent experiments. Scale bar, 20 µm. (b) Infected HUVEC 4 days after infection. Data shown represent the mean ± SD of three independent experiments. (c) Infected A549 cells 4 days after infection. Data shown represent the mean ± SD of three independent experiments.

Supplementary Figure 2.

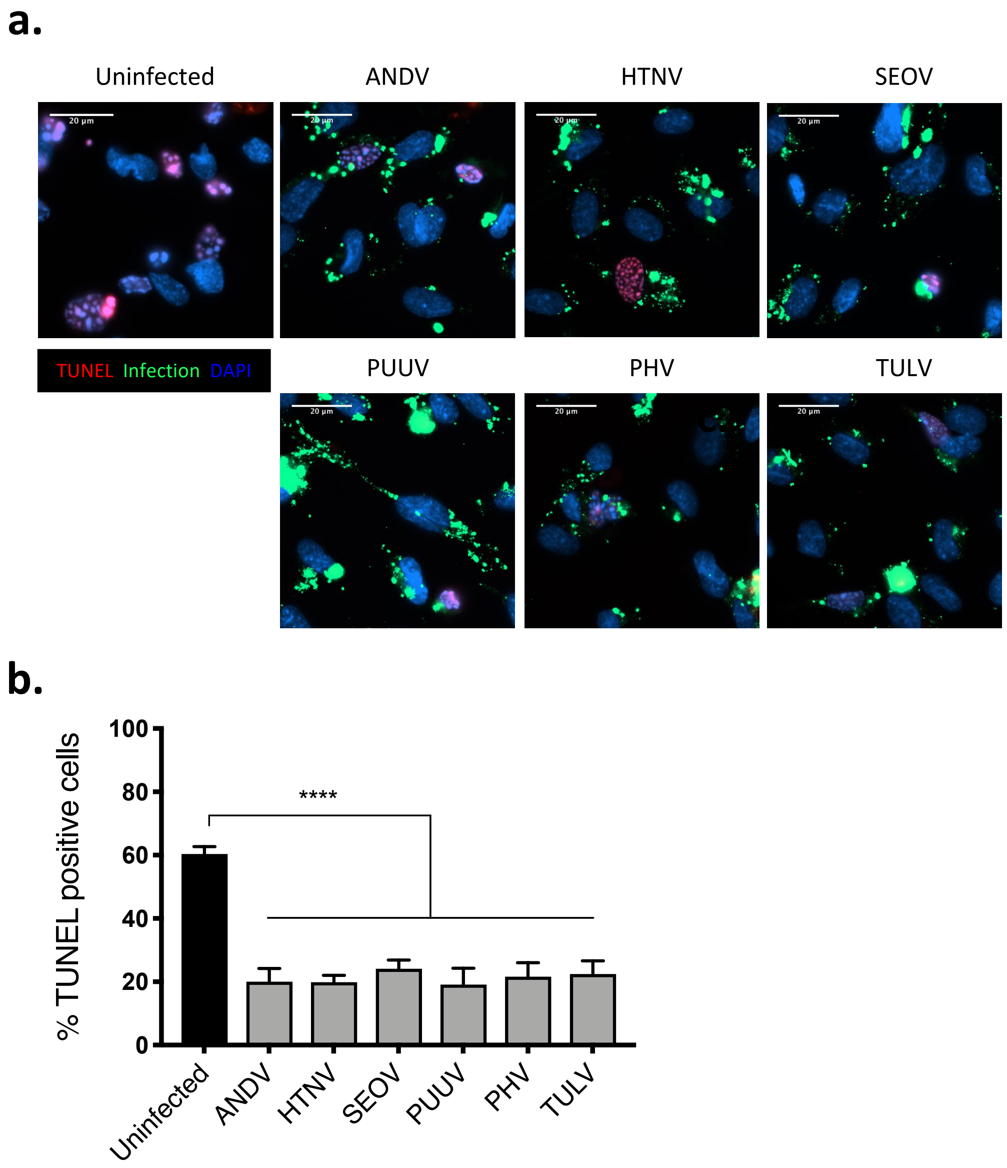

Supplementary Figure 2. Orthohantaviruses hamper staurosporine-induced apoptosis in cell cultures were almost 100% of the cells are infected. (a) Representative immunofluorescence images from three independent experiments of uninfected HUVEC, and ANDV-, HTNV-, SEOV-, PUUV-, PHV- and TULV-infected HUVEC after exposure to staurosporine (2 μM) for approximately 4 hours. Cells were infected at MOI 1 achieving ≥95% infection at 4 days post-infection. Following staurosporine-treatment, cells were stained with TUNEL (red) to assess apoptosis, convalescent PUUV patient serum (green) to detect virus infection and DAPI (blue) for nuclear counterstaining. Scale bar, 20 μm. (b) Graph showing TUNEL-positive uninfected and infected cells. Data shown represent the mean ± SD of three independent experiments.

Supplementary Figure 3.

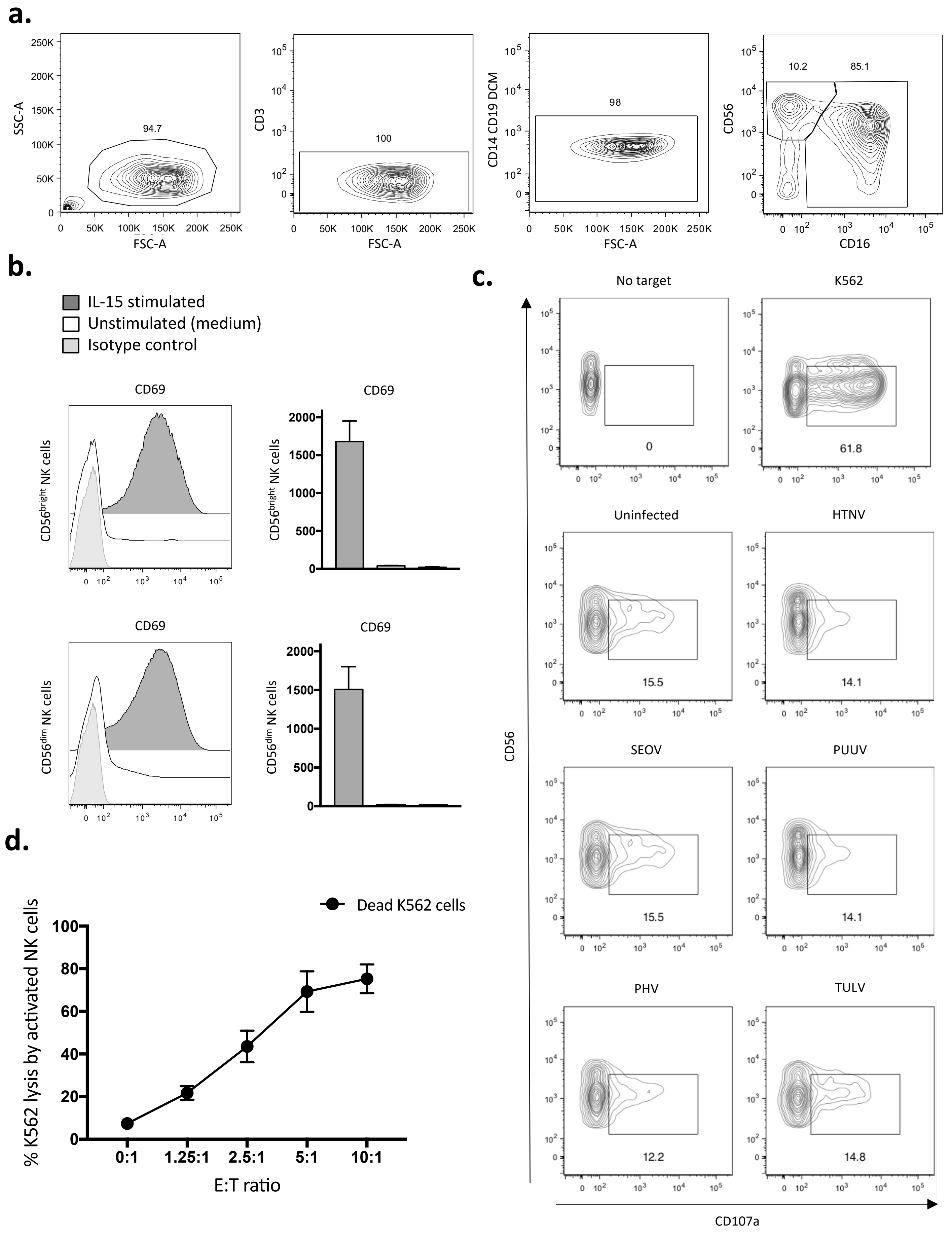

Supplementary Figure 3. NK cells gating strategy, analysis of NK cells activation after IL-15 stimulation and cytotoxic capacity towards K562 cells (a) Representative flow cytometry plots showing the gating strategy on NK cells: after size exclusion of small events, CD3 negative cells were selected, then dead cells and cells positive for CD14 and CD19 were excluded. Final gating shows NK cells as $CD56^{bright}$ $CD16^{negative}$ and $CD56^{dim}$ $CD16^{positive}$ cells. (b) Activation of NK cells by IL-15. Expression levels of CD69 (MFI) on gated $CD56^{bright}$ and $CD56^{dim}$ NK cells after over-night IL-15 stimulation. The graphs show mean ± SD of three independent experiments of three donors each. (c) Representative flow cytometry plots showing CD107a expression on IL-15 activated NK cells not exposed to target cells or exposed to orthohantavirus-infected or uninfected, HLA-blocked, endothelial cells for 2 hours. K562 cells were used as a positive control of NK cell degranulation. (d) Frequencies of dead K562 cells after exposure to increasing ratios of activated NK cells. K562 cells were CFSE labelled and co-incubated with NK cells for 4 hours; subsequent quantification was performed by flow cytometry with DCM. The results shown represent the mean ± SD of three independent experiments from nine donors in total.
